# Older Adults’ Perspectives of Independence Through Time: Results of a Longitudinal Interview Study

**DOI:** 10.1093/geront/gnad073

**Published:** 2023-06-18

**Authors:** Emily Taylor, Victoria A Goodwin, Susan Ball, Andrew Clegg, Lesley Brown, Julia Frost

**Affiliations:** Faculty of Health and Life Sciences, University of Exeter, Exeter, UK; Faculty of Health and Life Sciences, University of Exeter, Exeter, UK; NIHR Applied Research Collaboration South West Peninsula, Faculty of Health and Life Sciences, University of Exeter, Exeter, UK; Academic Unit of Elderly Care and Rehabilitation, University of Leeds, Bradford, UK; Academic Unit of Elderly Care and Rehabilitation, Bradford Institute for Health Research, Bradford, UK; Faculty of Health and Life Sciences, University of Exeter, Exeter, UK

**Keywords:** Autonomy and self-efficacy, Life course/life span, Resilience, Person-centered care

## Abstract

**Background and Objectives:**

Understanding how older people experience independence has implications for person-centered care. Existing understanding of older people’s experience of independence, generated through methods that provide a “snapshot” view of a person’s independence at a given time point, provides little insight into the process of maintaining independence through time. The aim of this study was to explore older participants’ perceptions to understand the processes and resources that were most important for maintaining independence.

**Research Design and Methods:**

Two semistructured interviews were conducted longitudinally to explore the perspectives of 12 community-dwelling, older people, aged 76–85 years. A social constructivist approach, using dramaturgical and descriptive codes, facilitated the interpretation of the data. Sixteen analytical questions guided the exploration of participants’ perceptions of independence through time.

**Results:**

Older people suggested that objective representations underestimated, and omitted, important aspects of their independence through time. Some participants perceived “snapshot” judgments of their independence as insensitive to their individual values and context. Change over time required some participants to adapt their methods for maintaining independence. The stability of participants’ sense of independence was value dependent and informed by the purpose a participant ascribed to maintaining independence.

**Discussion and Implications:**

This study augments the understanding of independence as a complex and multifaceted construct. The findings challenge the congruence of common interpretations of independence with older people’s views, showing areas of commonality, and discrepancy. Exploration of independence in terms of form and function provides an important understanding of how function takes precedence to form in determining the maintenance of independence through time.

## Background and Objectives

Many older people highly value their independence (Yuen et al., 2007) with the desire to maintain it outweighing other needs (Dunér & Nordström, 2005). Individuals go to great lengths to achieve independence, sometimes to the detriment of other aspects of well-being (Plath, 2008). Despite these potential negative consequences, the desire for independence persists at an individual and cultural level (Secker et al., 2003) and is reflected in health and social care policy (Department of Health, 2005; Department of Health and Social Care, 2021; Plath, 2009). Key to being able to continue living engaged and meaningful lives as people age, independence is also an important contributor to effective self-management of health conditions (Killingback et al., 2022) reducing demand on health and care services whilst empowering the individual to have agency in their care (Abdi et al., 2019).

Facilitating independence is challenging. Definitions of independence are many and interpreted differently between stakeholders (Davies et al., 1997; Secker et al., 2003). Independence is also an amorphous and shifting concept that can be both stable and dynamic through time (Parry et al., 2004). Maintenance of independence, therefore, has a temporal dimension, but the current understanding of independence through time is limited (Allam, 2015). Quantitative measures of independence based on the ability to conduct an extended range of activities of daily living (ADLs), provide a means to observe change in functional independence through time in health and social care settings (Åberg et al., 2005). However, less is known about older people’s views on, or priorities for, independence through time. Qualitative studies with older people provide evidence of shifting perceptions of independence as an adaptive strategy to maintain independence through time (Åberg et al., 2005; Haak et al., 2007; Meza & Kushner, 2017). However, these studies are cross-sectional or involve short follow-up times following an acute transition, making it difficult to develop an understanding of the process of maintaining independence through time in community-dwelling older people.

Using longitudinal analysis to explore the findings of two rounds of interviews conducted 12 months apart, this study explores the temporal dimension of independence from the perspective of older people. From the first interviews, conducted between July and December 2020, we generated initial themes about what independence means to community-dwelling people aged 75+ living in England. We identified that the concepts of participation (engaging in meaningful life activities), autonomy (being able to make and act upon your own decisions), and control (having control over whether and how help is received) were shared concepts within diverse understandings of the meaning of independence. Extending our understanding through a second round of interviews (conducted between August and November 2021), we aim to challenge and refine the findings to create knowledge about how views and experiences of independence may change through time.

### Aims

The aim of this study is to understand how older people perceive independence and to what extent these perceptions may change through time. Further, the study aims to understand the barriers and facilitators that influence experience and perceptions of independence through time.

## Research Design and Methods

### Design

A longitudinal qualitative design was selected to achieve the study’s aim of understanding how independence is experienced and the extent to which subtleties in personal, interpersonal, and contextual influences can enhance or hinder the experience through time (Green & Britten, 1998). Engaging a temporal lens for the analysis of two interviews conducted with each participant one year apart, enabled a deeper understanding of the breadth and depth of older people’s experiences and whether such experiences changed over time (Saldaña, 2002).

### Theoretical Framework

Social constructivism (Dupré, 2004) with a subjectivist epistemology provided the theoretical perspective for this study. As such, the authors contend that meanings are co-created between the participants and the researcher and that multiple different interpretations of meaning may be constructed, each of which are equally valid. The social aspect of this perspective speaks to the assumption that, though individuals are active agents in the generation of understanding, their meaning-making processes are dynamic, and influenced by interactions between themselves and their social contexts (Mercadal, 2020).

### Participants

Participants were recruited in 2020 from the UK-based Community Ageing Research 75+ (CARE75+; Heaven et al., 2019) cohort. When participants consented to CARE75+, they provided optional consent to be approached about future studies. Purposive sampling (Green & Thorogood, 2018) aimed for maximum variance in the characteristics of gender, age, living circumstances, and rural or urban location. Six participants were recruited by postal invitation with telephone follow-up, and a further eight were recruited through an open invitation distributed through the CARE75+ newsletter (Heaven et al., 2019). Participants gave informed consent to take part in the longitudinal study. Supporting documentation was sent to participants before confirming that they consented to continue with the study.

### Data Collection

The lead author collected data via telephone or video call depending on each participant’s preference. To achieve the longitudinal scope of this study, two interviews were conducted approximately 12 months apart with each participant. The analysis of the second interviews, conducted between August and November 2021 is the focus of this paper. Both researcher and interview participants engaged in the interviews from their own homes. Other members of the participant’s household were invited to attend the interview at the participant’s discretion (Polak & Green, 2016). The analysis was restricted to recruited participants’ comments only. Reflexive memos helped to consider, and provide transparency about, any perceived influence interpreted from the presence of non-interviewees (Saldaña, 2016).

A semistructured interview guide (Green & Thorogood, 2018) was developed by the lead author, then reviewed and refined during the discussion with the research team. The lead author informally piloted the guide (Ritchie & Lewis, 2006) through individual discussions with members of the projects’ Patient and Public Involvement and Engagement (PPIE) group. PPIE suggestions informed amendments to the guide, which improved the clarity and relevance of the questions. Following the first three interviews, detailed interview summaries (Miles et al., 2014), portions of transcripts, and initial thoughts about analysis, were shared with the research team. Similar iterative cycles of data collection and reflection were repeated and improved the credibility of the analysis through the consideration of different views and alternative interpretations (Green & Thorogood, 2018). Interpretive differences were incorporated, or resolved, through discussion.

Interviews were audio-recorded and lasted around an hour (range 38–90 min). Transcription of the audio recordings was shared between an external transcriber and the lead author, who also checked all transcripts for accuracy. Field notes (Saldaña, 2016), were made, recording details of the recruitment and interview process, and used to add depth to interpretation by considering the context of data generation.

The development of meaning is influenced by individual values and experience through the life course (Heinz et al., 2023). Therefore, rather than sampling for saturation, or “the point at which no new information or themes are observed in the data” (Guest et al., 2006), which may be impossible given the diversity of experience within older age groups, the sample size was determined through consideration of the implications of the study design on information power (Malterud et al., 2016). Several of the conditions that Malterud et al. (2016) associate with increased information power were met within the design of this study. For example, clear research questions and theoretical engagement were integral in the planning of the study, and pilot interviews helped to enhance the quality of the dialogue and to generate data with sufficient information power to obtain an in-depth understanding of how older people perceive independence through time. The relatively heterogeneous sample of older people obtained through purposive sampling introduced some uncertainty within the study design. Based on existing literature in which 6–12 interviews facilitated the generation of a comprehensive understanding of the meaning (Creswell & Creswell, 2018; Hennink et al., 2017), and from the experience of the research team, we determined that the sample size of 14 participants from the original study was likely to be effective in generating the desired information power. We reviewed and confirmed this decision before concluding data collection.

### Data Analysis

Deductive codes based on the International Classification of Functioning (ICF; World Health Organization, 2002) and descriptive codes related to the research questions (e.g., the meaning of independence) were used to organize data and enable comparison against similar organization of the original interviews. Temporal questions and associated coding processes (Saldaña, 2002) facilitated the analysis of change, enabling interpretation of patterns, and rhythms through time. Dramaturgical coding (Saldaña, 2016), which explores participants’ objectives, conflicts, strategies, and attitudes, was used to explore individual tactics and motivations toward maintaining independence through time. The use of dramaturgical coding methods was a strategic choice to fully explore the “roundness” (Donmoyer & Yennie-Donmoyer, 1995) and poignancy of personal experience evident from the initial interviews. Whilst deductive codes provided evidence of change between one point and another, and Saldaña’s (2010) 16 questions guided conscientious exploration of the shape and dimensions of change, the dramaturgical approach offered a method to generate a depth of understanding about how and why changes occurred at an individual level. NVivo 20 software (QSR International Pty Ltd., 2020) aided the organization of the data, memos, and field notes.

Themes were developed iteratively as the researchers proceeded with analysis alongside data collection. Prompted by insight generated through structured reflection between interviews, the lead author explored patterns and relationships between codes (Saldaña, 2016). Early interpretations were noted to reduce the risk of omitting important insight whilst keeping a transparent record of interpretive steps to facilitate reflection and scrutiny. As the analysis proceeded, the researcher viewed earlier insights through the lens of new interpretations generated by the researcher to see if they augmented, contrasted, or were separate from earlier understandings. Initial interpretations were then refined or rejected depending on their ability to facilitate a developing understanding. Members of the research team were involved in this process through regular meetings where the lead author shared their interpretations so far and the data that had contributed to them.

### Research Team and Reflexivity

Systematic and self-conscious approaches to each stage of qualitative research from design to communication are important strategies for ensuring quality, rigor, and therefore trustworthiness of qualitative research (Pope & Mays, 2006). Regular meetings with the multidisciplinary research team during the analytic phase of the study brought insight from different disciplines, experiences, and professional backgrounds. Discussion of the data and initial themes with the team increased rigor by stimulating dialogue and introducing alternative explanations that could challenge initial conclusions (Berger, 2015). Aware that researcher values are inevitably part of the research process (Green & Thorogood, 2018) the lead author welcomed the opportunity to dialogue with alternative perspectives and challenge potential biases or assumed knowledge associated with her background in physiotherapy. Keeping a reflective diary (Saldaña, 2016) enabled the researcher to continue this dialogue outside of research meetings, providing a transparent and self-conscious approach to managing potential biases and their impact on the research (Berger, 2015).

### Ethical Approval

This study was granted ethical approval by the University of Exeter Medical School Research Ethics Committee (RG/CB/20/03/243). The CARE75+ study was approved by the NRES Committee Yorkshire and Humber—Bradford Leeds on October 10, 2014 (14/YH/1120; Heaven et al., 2019).

## Results

Fourteen participants were interviewed in 2020; 12 had second interviews in 2021. One female participant died before the second interview. Despite several attempts, it was not possible to re-establish contact with the oldest participant in the original interviews, a 98-year-old male. Demographic characteristics of the included participants are provided and pseudonyms are used throughout to maintain confidentiality (Table 1). There were seven female and five male participants, and all were White.

**Table 1:** Demographic Details of Participants Interviewed in the Longitudinal Study

Pseudonym	Sex	Age (years)^a^	Living status
Bert	Male	78	Lives with spouse
Arthur	Male	81	Lives alone
Lucy	Female	78	Lives alone
Rose	Female	84	Lives alone
Jean	Female	79	Lives alone
Henry	Male	77	Lives with spouse
Tony	Male	80	Lives with spouse
George	Male	84	Lives with spouse
Monica	Female	83	Lives alone
Joy	Female	76	Lives with spouse
Margaret	Female	84	Lives alone
Nancy	Female	82	Lives alone

^a^At time of first interview.

Through our analytical approach, we generated three themes. The first two themes illustrate how the older people interviewed made sense of the different ways of conceptualizing independence when exploring their understandings in relation to common assessments of independence. The third theme speaks to the ways in which participants attended to the temporal aspects of independence within their interviews. Drawing on participants’ approaches to maintaining independence through time, the researcher details the two distinct concepts of *form* and *function* to illustrate how some participants can maintain a fulfilling experience of independence even when the form (or observable manifestation) of that independence has changed considerably.

### Theme 1: Activities of Daily Living Are Part of Independence But Set A “Pretty Low Bar”

Elaborating on a finding in the original interviews (under review), independence consistently meant more to participants than the ability to complete basic ADLs. In contrast to the original interviews, ADLs were referred to more frequently in participants’ meanings of independence in the longitudinal interviews but were insufficient to fully elucidate participants’ expectations of independence. Lucy’s response was illustrative of the need to “do a little bit more” than everyday tasks for her interpretation of independence.

I think being able to do things you’re interested in is important … I’d like to go to play bowls and … to take up a sport or something as well as [being] able to … wash yourself, dress yourself and do your shopping etc. … but do a little bit more. (Lucy, 78 years)

ADLs have a fundamental role in Lucy’s understanding of independence but are superseded by a need for greater engagement both cognitively and physically in situations that are meaningful to her. The increased reference to ADLs noted in participants’ longitudinal interpretations of independence may have reflected a restricted scope of independence experienced by nearly everyone during coronavirus 2019 (COVID-19). Rose talked about how lockdown restrictions and continuing concerns about travel and public events, altered what was forefront of her mind. In the first round of interviews, holidays were a consistent theme in Rose’s perception of independence, but their relevance had diminished over time due to restrictions.

Well, yeah, they are, they were, they [holidays] were [important] … Of course I haven’t been since … sometime during 2019 … I think the less you go [on holiday] ... they don’t seem to matter as much. … The brochures come through the door and I’ve lost my interest they usually go straight into the green bin. (Rose, 84 years)

Despite the restrictions, participating in life experiences that brought joy and meaning remained an important part of participants’ independence, with many returning to such activities as soon as possible or looking forward, sometimes longingly, to when they would be able to engage in them, or more of them, again.

Participants’ views on the use of an extended ADL scale to measure independence added to the conclusion that ADLs were only one part of a greater whole of independence. Joy described this way of measuring as “setting a pretty low bar really” and Nancy agreed that if it got to the point of considering ADLs then for her, “independence would have … almost gone” implying that the insensitivity of this measure would make it too late in detecting a decline in independence.

I think it’s setting a pretty low bar really .… I think being able to do things you’re interested in is important and if that’s … playing football or something it would be very difficult at a time like this [with COVID-19 restrictions], but it’s not for me. Being able to use your brain, being able to use skills ... (Joy, 76 years)

George’s interview indicated that although he was contributing more to his own IADLs (cooking and cleaning), he also indicated that he did not necessarily feel more independent. After a career in the building trade, he was no longer physically able to participate in many of his previous activities. This appeared to be difficult for George and may have contributed to his waning sense of independence.

I do feel upset sometimes about my [health condition] because I can’t physically do anything … When my wife is doing things that I used to do (,) that upsets me a bit. (George, 84 years)

That George remains independent in many ADLs, whilst simultaneously reporting a loss of independence reinforces the idea that independence requires more than ADL performance.

### Theme 2: Measures Out of Context Miss Important Nuances of the Lived Situation

To understand participants’ views of independence further and how they may be juxtaposed against common quantitative interpretations of independence, participants were invited to comment on findings obtained from a quantitative study (under review) designed to identify predictors of independence over time. Introducing this aspect of the interview brought into question the value of measuring independence based on specific, predetermined functions. This theme encompasses participants’ assertions that independence and its precipitators are highly dependent on the individual and there is limited value in measures that do not account for individuality. For example, Bert argued that trying to capture the essence of independence through a list of activities (as in quantitative measures of independence) was “wrong.” Independence was not just about the ability to complete an activity but about the ability to complete activities that were of value to, or on the “wish list” of, the individual.

It depends if these tasks are on your wish list … So, it’s the questions that are wrong ... I think if you have a list, you will never finish up with a list that suits everybody … So, maybe your list needs to be bigger and you need to choose certain items out of that list for specific people (Bert, 78 years)

Both former health professionals, Monica and Henry also disagreed with the idea that independence could be understood through a checklist and without a fundamental element of interpersonal interaction. Both feared that crucial information about the individual and their context could be lost when assessed through an impersonal “tick box” approach.

I was a [health professional] and if someone came in and said … could they talk to you … You could tell from seeing them and the way they reacted, [vs in] an e-consultation, you’ve only got the answers you can put down to the questions that are online. That doesn’t work. (Monica, 83 years)

If assessments are not [conducted] in a face-to-face scenario (,) I am not quite sure how they can relate health in old age to the conditions in which you have become accustomed to living. The world in which you live. In other words do you go down the pub every night? Do you rely on stuff like that to keep you sane? Or, do you do all the house stuff cleaning and gardening and all the stuff that occupies the mind? ... If you did nothing but tick boxes … you wouldn’t get the same impression. When you fill in a survey, no matter how many times you fill it in you are depending on somebody else describing what you would better appreciate if you saw it yourself. (Henry, 77 years)

The context was particularly important for understanding Henry’s perception of independence. To describe the discrepancy between how his independence would be measured and how he experienced it, Henry termed the former “your” independence deeming it the construct of independence of most interest from the academic/clinical view. Henry’s physical health had improved during lockdown and Henry argues that, by our standards, this made him more independent. On the contrary, due to difficulties within the family, Henry described his experience of independence as “compromised.” Corroborating this point, according to “our” measure of independence based on basic and instrumental ADLs, Henry had the highest possible score at every assessment point. The difference between his quantitative score and narrative reality demonstrates the chasm that can exist between what is measured and the lived situation.

Some participants did not question the measures. Though Tony acknowledged that “everyone is different,” he could see a benefit in standardized assessments to build up a picture of an individual’s health over time and therefore inform the support that might be needed. Jean supported her assumption that the assessments used must be effective and useful, by placing trust in the system not to waste time or resources without good reason.

Yes, I assume so … [that assessments in health and social care are helpful] they would be a bit of a waste of time otherwise ... I don’t really know specifically what [professionals] use [assessments] for but I assume that it is something useful. (Jean, 79 years)

### Theme 3: The Form Independence Takes Can Change, So Long As It Fulfills the Same Function

Using 16 analytical questions, developed to explore change through time in longitudinal studies (Saldaña, 2016), we interpreted the trajectories of change for each participant. In terms of physical and/or environmental change, some participants encountered sudden changes or “critical incidents,” such as hospitalization or sudden decrease in familial support, which marked significant disruption to their trajectory. Some experienced a more subtle decline through time, whilst some people’s physical abilities showed very little change. Significantly, the way that participants described their independence, did not necessarily follow the same course. Arthur was asked about independence and his experience of pain (observed from assessment responses). He described that whilst physical health could be quite volatile, with daily ups and downs, independence was much more stable and resistant to change even when achieving it became more difficult.

[My level of pain doesn’t really affect my independence, whereas] infections make things more difficult but it doesn’t really stop me doing what I want to do. (Arthur, 81 years)

When an individual’s subjective or objective view of independence did change it was not always obvious why that change had occurred. This was particularly evident when comparing the consecutive interviews of two female participants. Rose and Lucy are both widows, living alone, and both valued their sense of independence in the first interviews. Between February and August 2021, both experienced a series of “critical incidents.” Rose had a knee operation and was just recuperating when she collapsed and was hospitalized before being fitted with a pacemaker. Lucy described having a “painful year” resulting from a frozen shoulder, which persisted until about a month before the interview. On top of this, Lucy had been diagnosed with cataracts and described feeling as if “someone had turned on a tap” and drained all her energy. By the time of their interviews in autumn 2021, Lucy and Rose described themselves as approaching normal again after a period of recovery. If drawn on a graph, their physical trajectories would look quite similar, starting relatively high with a sharp drop followed by a gradual slope back up toward the starting level of health. However, despite similarities in their physical trajectory, their trajectories of feeling independent appeared to have divergent paths. When asked about changes in independence over time, Lucy’s tone changed immediately from a downbeat tone when talking about the physical difficulties of the last year, to one of excitement and positivity. Her physical experience seemed quite detached from her experience of independence. For Rose, however, the two experiences were clearly linked. Describing little change in the support she was receiving compared with the previous year, Rose still marked herself several points lower on a 0–10 independence scale because she did not feel as able.

*Interviewer*: So, last time you said that you had the gardener and things but you put yourself at seven or eight for independence out of ten. Do you think that’s changed at all?

*Respondent*: Marginally…Probably about five to six … I think at the present moment because of the back … I find I can’t … walk for as long as I could... Hopefully that will eventually get back to normal. (Rose, 84 years)

The difference in perceived impact of the critical incident on their independence could be due to differences in severity of the different afflictions. However, psychological impact also contributes to severity so it may not be appropriate to judge severity on physical impact alone. The way that Rose and Lucy described the meaning of independence in their first interview suggested that independence provided a different function for each woman. To Rose independence meant being able to do things herself and avoid reliance on others; to Lucy, independence was about managing on her own and making decisions following bereavement. As the critical incident impacted Rose’s ability to do things on her own and to the standard that she would wish, the function of her independence was compromised, and she felt less independent. However, the critical incident did not affect Lucy’s ability to make decisions and she was still able to manage on her own using compensation strategies, so she continued to achieve the function that she associated with independence leading to an unthreatened and unperturbed sense of independence.

Bert provided another example, which supports the idea that, so long as independence serves the desired function, the form it takes can change through time. Bert acknowledged that, in response to his spouse’s recent operation, his priorities in terms of tasks and activities had changed. Having spoken a lot about the importance of participating in hobbies that kept him physically and mentally stimulated for his sense of independence in the original interviews, Bert described the change in priorities not as a change in independence but as a “change in emphasis.”

I think when you retire you do things that you want to do … and then when something changes, you do things because you have to do them. So there’s a change in emphasis. (Bert, 78 years)

Thus, Bert feels that he is still achieving independence and the function he ascribes to independence, that is, keeping mentally and physically active, despite the change in the form that this has taken. However, it was clear that Bert understood this change in emphasis as a temporary state, from which he would return to his favored activities once his wife was well. As illustrated in the quotations below, the household tasks were much less mentally stimulating than the activities Bert usually enjoyed. Therefore although suitable for temporarily fulfilling the function that Bert required of independence, which was to keep mentally and physically active, their ability to fulfill that function longer term may be compromised.

*Interviewer*: … where you said about you were doing more housework …. how does that relate to your independence … compared to what you were doing before?

*Respondent*: Well, it was never in my list of duties. My list of duties were for the outside ... Just the fact that I prefer working outside so, doing house jobs is a chore. (Bert, 78 years)

## Discussion

Our rigorous analytical approach enabled us to identify that, whilst everyday changes in a person’s physical and physiological environment charted an unpredictable and fluctuating trajectory, individual understandings and experiences of independence remained relatively consistent through time. External pressures sometimes altered the activities (form) through which a person could achieve their independence but, if the new activities continued to facilitate the desired function of independence, then a sense of independence was maintained. We identified that preserving the function, and not necessarily the form, of independence was key to maintaining independence through time. Despite the individuality in interpretations of independence, the functions that participants ascribed to independence consistently extended beyond the ability to complete basic or instrumental ADLs. Of fundamental importance to care providers, by looking beyond the label of independence and through a longitudinal lens, we have conceptualized independence as a duality made up of form and function. “Form” refers to the observable activities, circumstances (e.g., living alone), and behaviors (e.g., accepting help) that are often used to indicate independence (Allam, 2015; Dunér & Nordström, 2005; Hammarström & Torres, 2010), whilst ‘“function” refers to the role or purpose independence plays for the individual older person such as enabling them to maintain a valued identity (Breheny & Stephens, 2012) or fulfill a vital social role (Narushima & Kawabata, 2020). The differentiation between “form” and “function” is commensurate with the concepts of “being” and “feeling” respectively which have aided the understanding of frailty (Grenier, 2006) and independence (Hammarström & Torres, 2010) in cross-sectional studies. Our choice to use “form” and “function” aims to avoid the implication that either is more “real” than the other.

The second aim of this study was to understand whether and how perceptions of independence changed over time. There is some ambiguity in the existing literature on independence as to whether it is a stable or dynamic concept. Differentiating the form from the function of independence deepens our understanding of how independence can be both changing and dynamic (Parry et al., 2004) whilst being perceived as remarkably constant throughout life (Åberg et al., 2005). Grounded in individual biography and intertwined with identity, we identified that “function” gave stability to individual meanings of independence. Honed through time, these expectations of independence were relatively persistent and resistant to change. In contrast, the activities, behaviors, and circumstances that contributed to the “form” of independence were much more vulnerable to external pressures and had the potential to be adapted to continue to perform the function required of independence (Åberg et al., 2005; Allam, 2015; Heinz et al., 2023). This highlights the need for a dynamic interpretation of independence. Through exploration of individual change through time, we explain existing discrepancies in the understanding of the temporality of independence by suggesting that the ability to maintain independence, and accept or adapt, following a change in activities depends on whether the new activities (form) are sufficient to maintain the purpose and function of independence crucial to its meaning for an individual. It is only when the form can no longer support the desired function that a sense of independence is diminished. This notion that stability and coherence gained from past experience can predict and help to navigate turbulence in the present and future is consistent with concepts of continuity (Atchley, 1989), coherence, and illness representations (Hagger & Orbell, 2003). The similarity between these models provides evidence that understanding independence would benefit from a life course perspective contrary to the cross-sectional and “snapshot” applications through which independence is widely interpreted in current practice.

Traditional ways of assessing independence through observation of its form, for example, the presence or absence of tangible outcomes, such as living in one’s own home, or ability to complete a task, neglects the importance of the role (or function) that independence has for an individual. Basing understanding of independence on the form it takes assumes that everyone will assign similar value to each activity that could contribute to independence. However, older people’s experiences are more nuanced, and whilst a particular outcome may mean loss of independence for one person, it may be not for another. Commonly used assessment scales, designed to aid operationalization, do not fully address this study’s participants’ understandings of independence, which went beyond everyday tasks of daily living. Nor were they sensitive enough to represent functions of independence that could be achieved despite limitations in ADLs, such as the ability to choose, or choose not, to participate in a given task or in a particular manner. Both of these results resonate with the findings of Ravensbergen et al. (2022). Rather than being a trait or characteristic that can be standardized, understanding the extent of a person’s independence requires attention to what that independence means to them experientially. The values and life-long commitments that define the function of independence for an individual are key to understanding the way a person chooses to live out their life and the motivations, and methods of problem-solving they will engage to do it (Kivnick & Murray, 2001). It provides an element of predictability making it possible for health professionals to work with an older person toward a shared vision rather than striving toward an assumption of independence that holds little meaning for the individual.

### Strengths and Limitations

A key strength of this study is that it adds a novel, longitudinal element to the understanding of independence, and indicates the importance of prioritizing the function of independence over its form, to achieve a truly person-centered orientation. The study involved participants from an existing cohort study, the double demand this imposed (i.e., to be willing and able to take part in two relatively intensive research studies) may have reduced the transferability of the findings due to a bias toward individuals who were fitter and more motivated than the wider population. However, at all stages, the potential implications of these limitations were considered and addressed as best as possible through recording and taking action on reflexive and analytical memos, researcher training, and consultation with public, and PPIE group members external to the study cohort.

Both sets of interviews took place within the first 2 years of the COVID-19 pandemic. The first interviews took place between July and December 2020 after the first U.K. lockdown had been eased in June. The introduction of a tiered system on October 14, 2020, meant that some of the Yorkshire participants were experiencing greater restrictions at the time of interview than participants in the South West. By the time of the second interview, participants had endured three national lockdowns but most legal limits on social contact had been removed. COVID-19 has implications for the transferability of the results as the atypical situation may have influenced participant’s frame of mind and therefore answers, especially in references to ADLs and IADLs which were restricted for everyone under lockdown rules. However, whilst the experience of lockdowns may have caused participants responses to be different from how they would have been in more typical circumstances, for many people the lockdowns highlighted personal values and vulnerabilities (Lewis et al., 2023) potentially strengthening rather than weakening the data.

## Implications

As several participants within this study argue, evaluating independence requires greater consideration of the individual, context, and values. Checklist tools or predefined questions which assume homogeneity in what people require or value for their independence fail to take into account individual contexts and risk leaving people feeling unheard and unsupported. Despite the difficulties in facilitating truly person-centered care (Killingback et al., 2021; Russell et al., 2002), our findings support the need to prioritize understanding of the individual and their context if we wish to facilitate independence that really matters to the individual (Department of Health and Social Care, 2021). As experts in their own experience of aging it is imperative that systems of support enable older people to be heard, involved, and pivotal in decisions about their care, improving their ability to self-manage and achieve better health outcomes as a result (Killingback et al., 2021).

## Conclusion

This study provides a novel interpretation that helps to provide an explanation for nuances in individual experiences of independence that cannot be understood through objective measures alone. Generating the concepts of “form” and “function,” we show how the experience of independence is determined by coherence between the “form,” or observable facets that can be employed to create independence, and the “function,” the role that a person ascribes to independence. Incorporating the strengths of person-centered care, we provide a tangible conceptualization that can practically inform communications and services aiming to facilitate the independence of older people.

## Supplementary Material

gnad073_suppl_Supplementary_Material

## Data Availability

The qualitative data collected for this study is not publicly available in the form of full transcripts to protect the anonymity of participants. However, examples of how the data have been interpreted to inform the themes of this study are provided in the Online Supplementary Material along with the interview guide. The study was not preregistered.
