# Supplementary material for: Older Adults’ Perspectives of Independence Through Time: Results of a Longitudinal Interview Study
Source: Gerontologist. 2023 Jun 18;64(2):gnad073. doi: 10.1093/geront/gnad073 (PMC10825833; doi:10.1093/geront/gnad073)
Supplement: gnad073_suppl_Supplementary_Material [file gnad073_suppl_supplementary_material.docx]

Online Supplementary Material

Interview Guide

**Opening Questions**

1. It has been about a year since our last interview, I hope that you have been well?
2. Last time we talked a bit about what independence means to you and what helps you to keep independent. I got the impression that at that time [*this will be personalised to the participant based on their responses in the first interview e.g. you felt that keeping physically and mentally active through your hobbies was really important for your independence*]. Does that sound right to you?
3. Did anything surprise you during or following that discussion?
4. Have you noticed any change to your independence in the last year? (this might be a physical change or a change in the way you think about it)

**Body of the interview**

1. First of all, I would like to ask, what do you think is the purpose of assessments (like those you do in Care 75+) in health and social care?
   1. What do you think about the use of assessments (general)?
   2. What do you think about the use of assessments specifically for independence?
2. Using statistical analysis, we found that: age, ability to perform basic activities of daily living, ethnicity, living circumstances, sight, frailty, cognitive function and depression seemed to be important predictors (positive and negative) of independence. What do you think?
   1. If Independence were a cake and these were the ingredients of the recipe, how well do you think the cake would turn out?
   2. Why? Are there any ingredients missing?
   3. Would you change the recipe depending on the characteristics of the person e.g. sex, married, multiple conditions, in hospital, care home, own home?
   4. What if the recipe was for successful ageing instead?
   5. How do you think autonomy would fit in? i.e. is it an ingredient, a separate cake or something else?
3. [*Relating responses to participants own data*] You mentioned/ we have talked about [*a predictor*] being important for your independence. I noticed that:
   1. At the (beginning) of the study your experience of [*predictor*] was a bit different to how it is now. Can you recall why you scored it differently and could you talk a bit about how that had an impact on your independence, if at all?
   2. Your score on [*predictor*] has been quite stable over the time you have been on the project.
      1. Can you remember a time when you might have scored differently? How was your independence at that point?
      2. Is there anything that you think could change that score? How would that affect your independence?
4. Finally, the COVID-19 pandemic and resultant restrictions, have had a big impact on us all. I’d like to know what, if anything:
   1. Has been a positive impact on your independence as a result of COVID-19
   2. Has been the biggest negative impact on your independence
   3. Would be the best thing that could be done to improve your independence from now going forward.
5. Is there anything else that you would like to add that we may not have covered or that you may have thought of since last year? Have there been any changes in that time that you would like to share with me?

### Derivation of Themes

| Key to coding method | |
| --- | --- |
|  | Descriptive codes based on research question |
|  | Deductive codes derived from ICF |
|  | Dramaturgical codes |
|  | Longitudinal Questions |

The coding diagrams below provide examples of how the primary data were interpreted through multiple perspectives facilitated by different coding methods to derive the final themes. The key shows which coding method was used to generate the code used.

| Theme | Code | Primary data in the form of participant quotation or analytical memo |
| --- | --- | --- |


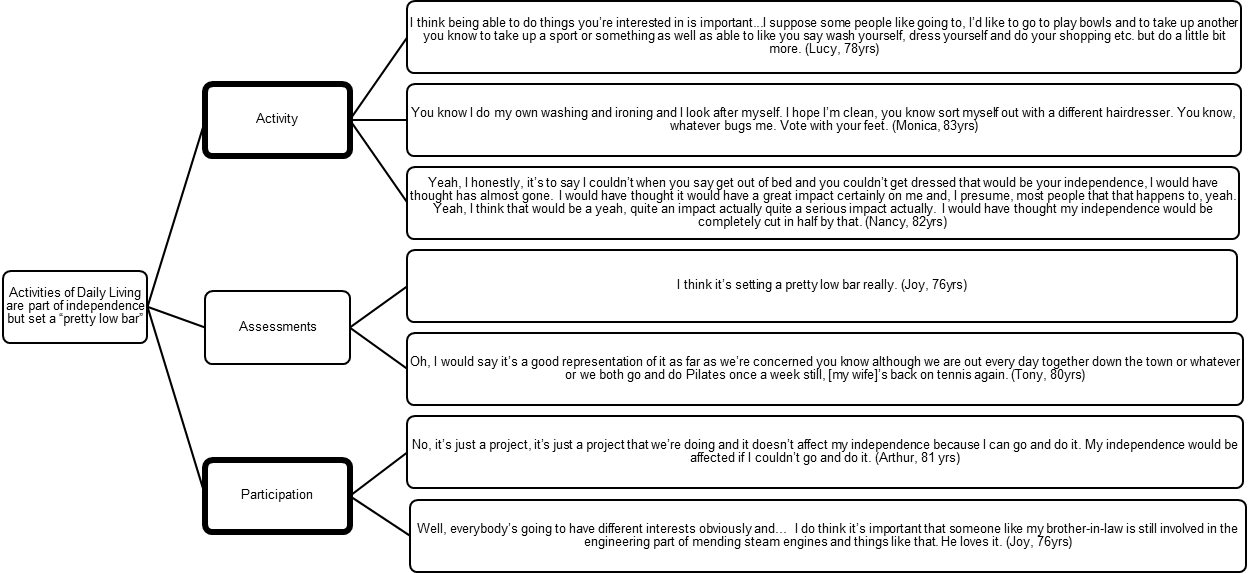


| Theme | Code | Primary data in the form of participant quotation or analytical memo |
| --- | --- | --- |


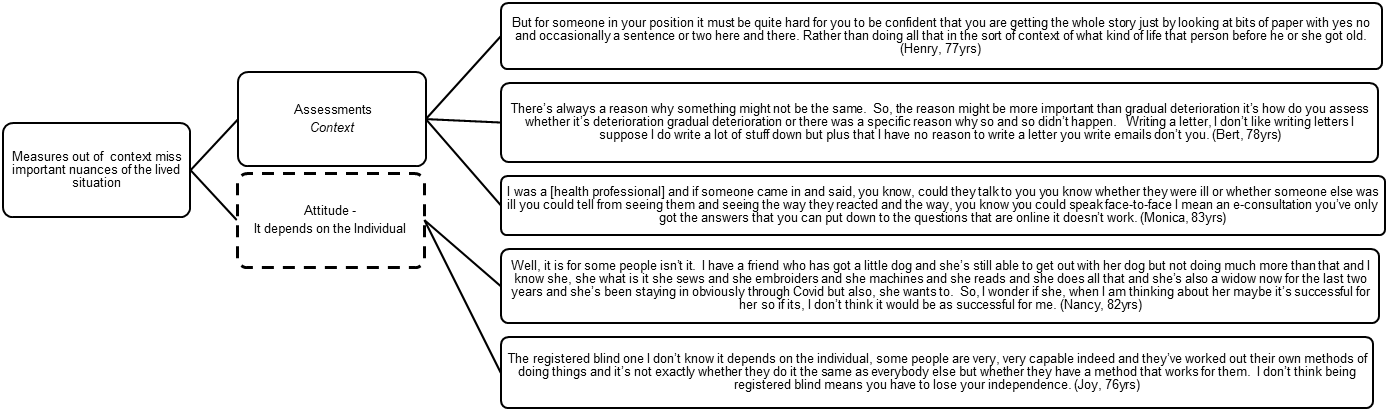


| Theme | Code | Primary data in the form of participant quotation or analytical memo |
| --- | --- | --- |
